# Is there a moral obligation to conceive children under the best possible conditions? A preliminary framework for identifying the preconception responsibilities of potential parents

**DOI:** 10.1186/1472-6939-15-5

**Published:** 2014-01-22

**Authors:** Pieter Bonte, Guido Pennings, Sigrid Sterckx

**Affiliations:** 1 Faculty of Arts and Philosophy, Ghent University, Blandijnberg 2, 9000 Ghent, Belgium; 2 Faculty of Arts and Philosophy, Vrije Universiteit Brussel, Pleinlaan 2, 1050 Brussels, Belgium

**Keywords:** Preconception care, Beneficence, Folic acid, Obesity, Genetic testing

## Abstract

**Background:**

The preventative paradigm of preconception care is receiving increasing attention, yet its boundaries remain vague in three respects: temporally; agentially; and instrumentally. Crucially, it remains unclear just who is to be considered a ‘potential parent’, how soon they should take up preconception responsibilities, and how weighty their responsibilities should be.

**Discussion:**

In this paper, we argue that a normal potential parent of reasonable prudence has a moral duty to adequately optimize the conditions under which she or his reproductive partner will conceive, though a proportionality calculus calls for toleration of several forms of preconception behaviour that are non-ideal from the perspective of reproductive risk. We distinguish between five categories of potential parents to which different duties of preconception care should be ascribed. This framework is advanced to assign preconception care responsibilities with more precision than is often done in the current debate on preconception care. We conclude by applying our theoretical framework to three types of preconception care interventions: consumption of folic acid; keeping one’s weight under control; and engaging in preconception genetic screening. Our analysis shows that the literature on preconception care often glosses over crucial distinctions between different types of potential parents and uses a notion of preconception beneficence that may be overly demanding. Nevertheless, preconception moral duties will often be weighty and reluctance to accept such duties on account of the burden they impose do not warrant preconception insouciance.

**Summary:**

To avoid misplaced responsibility ascriptions in the growing field of preconception care, distinctions must be made between different types of potential parents to whom different degrees of preconception responsibility apply. We present such a preliminary framework and bring it to bear on the cases of folic acid consumption, obesity and genetic testing.

## Background

According to the Health Council of The Netherlands, ‘preconception care’ (henceforth PCC) refers to the large cluster of interventions “aimed at ensuring that couples who wish to have children start a pregnancy under the best possible conditions” [1]. Though clearly demarcated at one end by the occurrence of conception, at the other end the boundaries of PCC can be vague in three respects: temporally; agentially; and instrumentally. Temporally, the concept of PCC can be understood to refer to all acts and omissions which might affect the good of future persons, which at the extreme include the acts and omissions of distant ancestors. Agentially, PCC can refer to a broad array of agents from ‘potential parents’ and all the subcategories thereof (see below) over myriad medical professionals to moral communities and political institutions. Instrumentally, the armoury of PCC can be stretched to include not only specific medical interventions and family planning but all kinds of acts and omissions that are instrumental in creating the best possible (or at least minimally decent) conditions in which to conceive future persons.

In this paper, we start by briefly sketching a variety of PCC measures that contemporary potential parents could engage in, thereby giving an idea of the large number of options currently available to conceive under optimal or minimally decent conditions. Second, we seek to provide a categorization of the ethically relevant types of ‘potential parents’. Third, we develop a normative argument about what the ethical principles of beneficence and nonmaleficence demand of potential parents. Finally, we apply the resulting general conception of potential parents’ preconception responsibilities to three cases: consumption of folic acid; avoidance of obesity; and undergoing screening for genetic risk.

## Discussion

### What can potential parents do?

The PCC-armoury available today contains a wide range of sufficiently effective, evidence-based interventions for potential parents to merit considering them [1]. For the purposes of this paper, it is sufficient to give an idea of the demands that a fully-fledged PCC regime would put on potential parents. They would be asked to: (1) follow a number of specific dietary prescriptions; (2) take specific supplements; (3) avoid obesity and anorexia; (4) moderate or abstain from use of alcohol, tobacco, and various other recreational drugs; (5) avoid specific environmental exposures and chemicals; (6) avoid excessive psychological stress; (7) take specific precautionary measures in case of maternal health problems or when taking certain forms of medication prior to conception; (8) avoid consanguinity and (in case of suspected significant risk) undergo genetic screening and, if necessary, take appropriate measures, such as using assisted reproduction techniques, choosing a different reproductive partner or abstaining from reproduction; and last but not least (9) time conception at an ‘optimal age’ via contraception and other means of family planning.

In regions with well developed health care systems, the incidence of many forms of adverse pregnancy outcomes has decreased dramatically throughout the 20th and early 21st Century. However, as the latest March of Dimes Global Report on Birth Defects shows, the incidence of birth defects remains considerable everywhere [2]. According to this report, worldwide, approximately 8 million children per year were born with a serious birth defect of genetic or partially genetic origin – i.e. 6 per cent of all births. In France, the country for which the March of Dimes reported the smallest number of birth defects, there were still 39.7 children per 1000 live births born with a serious congenital abnormality. Around the globe, human reproduction remains far from risk-free, and intensified PCC is one promising avenue to reduce human suffering. Moreover, the case for intensified PCC gains all the more urgency if one factors in the number of abortions which often entail psychological damage, physical pain, and also grave health risks to the mother when sub-optimally performed [3]. Many of these risks could have been avoided by better access to and use of contraceptives or by the adoption of additional PCC measures to improve the timing of the pregnancy and the viability and health of the child [3].

### Who is a ‘potential parent’?

A contemporary potential parent may be confronted with her or his (alleged) PCC responsibilities by at least three groups:

a) public health and child care providers who seek to enlist potential parents in their respective projects, as well as personal health care providers who provide directive counselling;

b) private for-profit providers of PCC interventions, such as direct-to-consumer genetic screening and counselling companies who have a commercial interest in creating demand for their services; and

c) particular moral communities (e.g. anti-abortion activists) who hold moral views that prescribe duties of PCC to potential parents.

However, it is often unclear exactly who these groups are targeting. At times, only prospective parents are being addressed (for instance in the above characterization of PCC by the Health Council of The Netherlands). At other times, the category of addressees is expanded to include everyone who is (presumably) fertile or is nearing fertility (see for example the recent proposal by the UK Human Genetics Commission to offer genetic screening during the final years of secondary education [4]). This shows that many different types of ‘potential parent’ can be identified to which very different degrees of responsibility might apply. In this section, we outline a categorization of potential parents in which a balance has been struck between precision and practicability. Our categorization roughly follows the lines of probability and intention to conceive, where ‘probability’ includes (presumed) capacity as well as behaviour. Despite first appearances, it does not necessarily reflect a linear temporal order. We distinguish the following five categories:

1) Prepubertals nearing fertility (no capacity, no behaviour, no intention).

2) Fertile persons who are not sexually active (or only non-coitally) (capacity, no behaviour, no intention).

3) Sexually active persons with no intention to conceive in the foreseeable future (capacity, behaviour, no intention). This category also includes persons who are duly compliant in their use of contraceptives, but whose contraceptives are not fully reliable.

4) Sexually active persons with an unclear intention, who wilfully abstain from contraception and leave it to chance/nature whether conception will occur or not (capacity, behaviour, intention unclear).

5) Prospective parents: fertile, sexually active persons who intend to conceive in the foreseeable future (capacity, behaviour, intention). This category also includes persons using assisted reproductive technologies.

Bearing these distinctive categories of potential parents in mind will help to avoid making category mistakes such as lumping together too many different types of potential parents when ascribing preconception duties of care to them and expecting them to meet those duties (possibly backed up with sanctions if they do not). However, in some forms of PCC awareness-raising, there may be good reasons to lump all potential parents together. For instance, one powerful argument for a non-stop stance of PCC prudence (for all potential parents) is the high incidence of unintended and ill-planned pregnancies. On some estimates, unintended pregnancies alone amount to 41% of pregnancies worldwide and remain prevalent in developed regions [3]. Indeed, in the categorization outline above, unplanned or ill-planned conception might occur in all groups who have the capacity to conceive and are sexually active.

### What should potential parents do?

The question arises, however, as to what constitutes ‘good planning’, and to what extent and on which grounds this can be morally demanded of potential parents. One possible ground is a duty of beneficence, i.e. a duty to advance the good (of others), often by active intervention [5]. Such a duty can be said to hold if not generally, then at least for persons with specific relational roles, such as a parent towards his or her (future) child. Referring to the work of Derek Parfit, Savulescu and Kahane observe that “in selecting a more advantaged child we are also bringing a different person into existence”. This poses a ‘non-identity problem’ as to “what might ground a moral obligation or reason to select such a child”. They go on to argue that one can nevertheless maintain the case for a moral obligation of procreative beneficence, for instance on *impersonal* grounds. As such, the reason to be beneficent “is that selecting the most advantaged child would make the outcome better, even if it is not better for the child created” [9: 277]. To illustrate with an abstracted clear-cut case with all other things being equal, if one can either put a ‘bundle of joy’ or a ‘bundle of suffering’ on the planet, there would be a strong moral obligation to conceive a joyous rather than a tormented child [9: 279].

Another possible ground is a duty of nonmaleficence, a duty not to harm others, often by passive abstention [5]. Nonmaleficence will often be less demanding than beneficence, but on the other hand it may be demanded of more persons, for instance universally and not only of those standing in some specific relational role. If some potential parent would only have to be nonmaleficent in relation to her potential future child, more leeway should be given to her own right to autonomy: she should then be free to live her life as she sees fit without being duty-bound to procure the good (for someone else). She should only refrain from harming others.

### Preconception beneficence - above all, do good towards one’s potential child?

Many contemporary ethicists would argue that the prime focus of reproductive decision making should be the wellbeing of the resultant child. To engage in PCC from the motive of unburdening or strengthening society or of satisfying the parents’ instrumental plans with regard to the child would be open to the same criticisms that have profoundly discredited the eugenic reproductive schemes prevalent from the end of the 19th Century up to the late mid-20th Century [6,7].

Having regard to prioritizing the child’s wellbeing, Savulescu and Kahane defend the following ‘principle of procreative beneficence’ (PB, first coined in [8]):

“If couples (or single reproducers) have decided to have a child, and selection is possible, then they have a significant moral reason to select the child, of the possible children they could have, whose life can be expected, in light of the relevant available information, to go best or at least not worse than any of the others” [9: 274].

Although the use of the phrase ‘procreative beneficence’ seems to suggest a principle relevant to all procreative issues, Savulescu and Kahane formulate the principle in a highly targeted way. For instance, they note that: “PB is silent on a number of further questions in procreative ethics [. For instance it] assumes that a decision to have a child has been taken.” [9: 274, footnote 3]. Their discussion is also focused on settings involving selection, in which one can make a choice between different gametes or embryos. Within the bounds of these constraints, Savulescu and Kahane have made a forceful argument that PB operates as a primary moral principle which will often override other principles in play such as procreative autonomy. In brief, they argue that procreative autonomy allows for parents to intentionally create a child who, for example, “will live a brief life of misery and torment” [9: 279] even when they could have alternatively created a child in good health. Savulescu and Kahane find such parental autonomy *morally* unacceptable as well as in violation of much common sense morality. That said, they do allow for parental autonomy to possibly remain a primary *legal* right. Moreover, they hold that, other things being equal, PB entails maximizing parental commitment to provide the best chance for the best possible life. Less far-reaching aims such as a ‘life worth living’ or a ‘disease and handicap-free life’ will not do.

In this article, we will not contest Savulescu and Kahane’s formulation of the principle, nor their application of it. Rather, we will take their principle as-is but remove the restriction of its application to prospective parents so as to find out what it would imply if applied in the preceding domains of preconception care. Rather than taking on board the additional question of ‘enhancement’ as Savulescu and Kahane do, in order to retain focus, we will not contest the conventional ethico-medical standard that the best condition to provide for future children does not go beyond a ‘normal’ state of disease- and handicap-free existence. As we do not provide a justification for a principle of PB, those who deny the existence of such a principle may also find our extension of that principle unconvincing. Alternatively, our extension of the PB principle may make the account offered by Savulescu and Kahane more compelling for some.

Interestingly, preconception care advocacy often (implicitly) appeals to PB, and this may corroborate Savulescu and Kahane’s assertion that PB has substantial commonsensical appeal. Nevertheless, we will argue that, in the domain of PCC, PB runs up against formidable competing concerns. This may be sufficient to cast significant doubt on the thesis that PB can play the role of ‘first principle’ in PCC. If this holds, contemporary PCC advocacy may need to fundamentally rethink certain awareness-raising campaigns and PCC counsellors their counselling practice.

To apply Savulescu and Kahane’s PB in the field of PCC, it would need to be rephrased along the following lines to constitute a ‘principle of preconception beneficence’:

“If one can take/refrain from action prior to conception to, in light of the relevant available information, significantly increase the likelihood that if one conceives it will be of a child whose life can be expected to go best or at least not worse than the lives of any of the other children one may otherwise conceive, then one has a significant moral reason to take/refrain from such action”.

If this would be the moral standard prescribed for all potential parents, they would have to face up to a long and taxing PCC checklist that will only lengthen as science and technology increase the range of preconception options that may serve to optimize reproductive outcomes. Moreover, persons at an ever-widening distance (in time or in intent) from conception may find themselves being drawn into the expanding sphere of PCC responsibility. Given that for instance the California Preconception Initiative advocates that women be made aware of PCC at every medical visit throughout the health care system, following the dictum “every woman, every time” [9], they might have to answer at every turn why they are not doing all they can, as soon as they can, to ensure that, should there be any future pregnancy, it will be a “pregnancy under the best possible conditions” [1].

The practical burden of long-term compliance with a complex set of prescriptions to ensure a good that may be very distant and/or improbable, is not to be underestimated. As Singh and colleagues write on the specific topic of contraceptive use:

“By the time she is in her mid-40s, a woman with two children will have spent, on average, only five years trying to become pregnant, actually being pregnant and not being at risk for another pregnancy for a few months following a birth. To successfully avoid becoming pregnant before, after or between those two births, either she will have had to refrain from having sex, or she or her partner will have had to practice contraception effectively for an average of about 25 years—a hard standard of behaviour to live up to, even for the most disciplined and highly motivated individuals” [3].

Although the use of contraceptives has by now (in the developed world at least) become a more or less accepted responsibility for the majority of sexually active persons, for all its blessings the effort of maintaining adequate compliance remains a substantial burden. To this burden, the PCC armoury invites us to add staying informed and up-to-date about the state-of-the-PCC-art, maintaining dietary and physical exercise routines, avoiding certain environments and toxins, undertaking medical screenings and check-ups, securing adequate rearing-resources (not only financial and material but also psychological, pedagogical, social and cultural) prior to conception, etc.

The mere (potential) availability of some effective PCC intervention is sufficient to impel a person to justify (if not to others, then at least to herself) why she would not make use of it. This can be experienced as a ‘technological imperative’, or more generally, as a ‘capability imperative’: as soon as some newfound mode of intervention is made available, one’s sphere of possible agency is expanded, and one inescapably finds oneself at liberty to influence states of affairs where one used to be factually impotent to do so. Any newfound power thus puts us at liberty to either use or not use it, thereby literally forcing a new responsibility on us.

As PCC advocates now call for pervasive and perpetual awareness-raising programs aimed at all potential parents [10], the risk arises that an increasing number of people become susceptible to criticism of being or having been a ‘failing potential parent’. Moreover, as the armoury of PCC and its availability expand, people become susceptible to such criticism to an increasing degree.

From the vantage point of preventative health care, there are good reasons to start assuming responsibilities of PCC as soon as one nears reproductive age. For instance, many of the effective PCC interventions are lifestyle and work environment changes, and such changes are only likely to have sufficient effect by the time conception occurs if they take place well before conception [1]. In a similar vein, lifestyle habits engaged in during one’s twenties are likely to become entrenched ways of living for the rest of one’s life, and altering one’s habits in later years is likely to require greater effort. Thus, as many may fail to muster sufficient intrinsic motivation to develop healthy habits and make healthy choices because the (moral) gratification is too uncertain and/or too remote, they may need to be prodded and incentivized by others in sufficiently early, constant and intensive ways.

A telling example of such a hands-on incentivizing campaign is the ‘Don’t U Dare’ PCC promotional video of the March of Dimes foundation [11]. In this promotional video in the scripted reality format, a PCC coach closely monitors a ‘merely fertile’ woman (category 3) and (cheerily) chides her for every suboptimal move she makes. Despite its superficial cheerfulness, this awareness-raising material seems saturated in an emotionally manipulative discourse of shaming and blaming and may therefore amount to a form of PCC counselling that is highly directive. Much the same seems to hold for the nation-wide ‘Show Your Love’ campaign of the US Preconception Health and Health Care Initiative and the California Preconception Initiative, which suggests to potential parents that if one does not engage in PCC, one may be lacking basic parental love [12].

In a more comprehensive analysis of PCC, as opposed to the preliminary assessment we are offering here, one should also scrutinize the extent to which today’s PCC awareness-raising campaigns may be (co-opted as) modern-day heirs to entrenched community traditions in which a girl’s identity is narrowly scripted as ‘future mother’ – a script of social expectation and obligation that can be enforced by playing to fears that if a girl or woman engages in athletic pursuits, takes on stressful studies or employment, for example, she might be endangering her central *raison d’être:* that of being a responsible ‘future mother’. To be fair, men are also being asked to engage in certain forms of PCC to optimize semen quality or to aid and support (and, perhaps, to coax and keep compliant) their female reproductive partner [13], yet overall their potential PCC responsibilities pale in comparison to those ascribed to women. PCC advocate Merry-K. Moos has engaged with the worry that PCC might “frame women as nothing more than vessels for growing healthy offspring” [9], and largely dismisses it. Commentators such as Rebecca Kukla, on the other hand, discuss the increasing and unreasonable burdens women are expected to accept on their way to becoming a mother [14]. In a similar vein, PCC is at risk of being co-opted in dubious practices of “hyper-parenting”, where competitive, perfectionist and over-anxious parents seek to control and plan ahead the lives of their (future) children to an ever increasing extent [15].

Messages entailing a substantial responsibility expansion for potential parents can also come from the very different corner of for-profit health care providers. For-profit entrepreneurs have a marked commercial interest in inflating notions of individual responsibility and fanning the flames of hyper-parenting: the more that potential parents believe themselves to be inadequate, and the more that people consider themselves to be potential parents, the greater the demand for the services of such entrepreneurs. In the world of direct-to-consumer genetic testing companies such as Counsyl and 23andMe, marketing techniques of commercial demand creation in the guise of public-spirited ‘awareness-raising’ seem to be standard fare [16-18].

Thus, for example, Counsyl, the for-profit provider of a highly media-hyped ‘Universal Test’ for genetic risk, highlights on its website the following quote of Professor Patrizio, director of the Yale Fertility Centre: “Every adult of reproductive age should consider the Counsyl test before pregnancy.” As Counsyl-CEO Srinivasan likes to envision it, his company’s test should not only be ‘universal’ in its testing capacity but also in its use: “one of our goals is to make this like the home pregnancy test” [19]. Occasionally such messages are taken to hyperbolic extremes. For instance, the director of the for-profit Centre for Surrogate Parenting and leading US radio host Bill Handel has opined that conceiving of a child via coitus has today become offensively irresponsible: “I always get astounded and offended when people actually have sex to have kids. I don’t understand that. They shouldn’t do that. You can always use some high-tech form of reproduction” [20].

Not only do such for-profit actors often severely overstate the moral obligation of potential parents to become PCC customers, they also tend to severely overstate the effectiveness of the services they market. Without proper policies to mitigate misinformation and manipulative ‘demand creation’, the general public will often not be able to distinguish between bona fide and not-so-bona fide players in the PCC field [16]. As a result, they are at risk of lumping all these responsibilizing messages together, thus creating a sense of PCC responsibility that is needlessly cumbersome.

Nevertheless, though Handel’s suggestion is grossly excessive given today’s state of the art, Savulescu and Kahane have argued that “[a]s means of selection become safer and our ability to use them to select non-disease characteristics increases, we believe that PB [procreative beneficence] will require most reproducers to select the most advantaged child unless doing so is predicted to lead to a very significant loss of well-being to existing people” [9:281]. This implies that, if assisted reproductive technologies would ever turn into full-blooded alternatives that are significantly less risky than natural reproduction, anyone who has access to such technologies would have significant moral reason to relinquish natural procreation altogether in order to reproduce in the safer, artificial way. Whether or not one objects to this specific example, the general point remains that simply by upholding the very same moral standard that governs today’s use of PCC, potential parents may find themselves morally obliged to engage in quite unsettling acts and omissions as PCC capabilities expand.

### Preconception nonmaleficence and the autonomy of potential parents

We now turn to some arguments which seem to provide legitimate, principled objections to the primacy of preconception beneficence. If these objections hold, they would relax the taxing demands of preconception beneficence discussed earlier.

Insofar as a ‘potential parent’ falls beneath certain thresholds of intent to cause conception and/or probability to cause conception, it becomes problematic if not outright incoherent to expect such a person to take up certain presumed role responsibilities of a parent. Since she would not fit the description of a parent or procreator, it would make little sense to ask her to fulfil particular parental or procreative duties. Indeed, to the extent that potential parents would not be parents, other principles can assert themselves, most importantly the principle of individual autonomy. In principle, such ‘non-parents’ should be free to lead their lives without being excessively constrained by concerns about the wellbeing of unintended and merely potential children.

This is not to say, of course, that non-parents would thereby be relieved of the general responsibility to avoid inflicting harm upon others, a duty that stems from the general principle of nonmaleficence [5]. This universal duty to do no harm, which is codified in some form in virtually all established moral theories as well as in civil law, applies to non-parents and parents alike. However, this universal duty of nonmaleficence obviously needs curbing, lest one is (absurdly) held responsible for all possible harm (no matter how minute) to anyone (no matter how remote). In order to properly apply the principle of nonmaleficence and to discern whether the corresponding duty is at play in a given situation, further stock concepts from moral philosophy and law need to be brought in [21].

For our purposes, it is sufficient to invoke the concepts of reasonable foreseeability, adequate control, adequately proximate causation, proportionality, and reasonable prudence:

1. Foreseeability (requiring adequate cognizance by the wrongdoer of the consequences of his act or omission);

2. Control (requiring adequate control by the wrongdoer over the events in which he was implicated);

3. Proximate causation (requiring that the act or omission of the wrongdoer was an adequately proximate cause of the adverse turn of events);

4. Proportionality (requiring that the benefits of the intervention are in proportion to the effort that must be invested to avoid the wrong). We will consider proportionality in relation to the standard of a ‘normal person of reasonable prudence’: preconception acts and abstentions that are disproportionately burdensome to such a person will not be morally required. Normally proportionality is calculated as follows: probability of an affliction in a future child x gravity of the affliction/cost of precaution. With regard to PCC, however, this calculus – already difficult to apply in a sufficiently precise and methodologically satisfactory way – is further complicated by the fact that the calculus must be made prior to conception, which can add great uncertainty and because one has to factor in the probability of conception, which is highly unclear in most cases. Thus, the calculus to be applied with regard to PCC takes the following form: probability of conception x probability of affliction x gravity of affliction/cost of precaution. This added complexity alone has caused certain judges to declare preconception torts inadmissible [21].

### Applications: folic acid, obesity, genetic testing

In order to more precisely assess the responsibilities of potential parents in specific cases of PCC, the general conception of preconception responsibility outlined in the previous section (‘Principles’) needs to be applied to specific PCC interventions and specific types of potential parents. In this section we will provide three brief casuistic illustrations to put our general conception of preconception responsibility to work: folic acid, obesity, and genetic screening. We will highlight where and why preconception responsibilities significantly increase or decrease between different types of potential parents.

### Folic acid

The potential suffering brought on by neural tube defects such as the gravely adverse condition of spina bifida is significant and the chance of such defects occurring is 1/1000 for American procreators [22]. A strong evidence base has been established, indicating that the consumption of folic acid supplements, for a period of about three months prior to conception, reduces by two thirds the risk of neural tube defects [1].

Given the framework of preconception responsibility outlined above, does this make it morally required for any normal, reasonably prudent potential parent to begin taking folic acid in due time?

Cognizance. One needs to be aware of the importance and possibility of achieving an optimal folic acid intake in order to be able to do so in a timely fashion. This requires education via awareness-raising campaigns, timely advice from GPs, obstetricians, etc. Unfortunately, even in countries such as The Netherlands, where efforts at widespread informational campaigns on folic acid have been made, many women remain unaware about the existence and importance of folic acid [1]. As things stand, this can hardly be blamed on a failure of these women to have solicited proper and timely advice on preconception care. This may surely change, however, once folic acid intake becomes a standard fixture within public health education.

Control. Provided that one has ready access to folic acid (financial, logistic and otherwise; conditions that may again not be met in many situations), the intake of this supplement is quite feasible and does not seem to be very demanding, neither as regards expenditure of money, time, or effort, nor endurance of side-effects (optimizing folic acid levels does not produce any negative side-effects for the mother-to-be).

Causation. Should one forego folic acid intake, this omission would become an important co-cause of (the higher probability of) eventual neural tube defects in future offspring.

Proportionality. A normal prospective mother of reasonable prudence can reasonably be expected to shoulder the very minor burden of taking folic acid tablets, and reproductive partners can equally be expected to support and stimulate their child-bearing reproductive partners to do so [13]. Even in the presence of multiple other demands and given the daily hustle and bustle of everyday life which can complicate proper compliance with prescribed medical routines, this does not impose an unreasonable or disproportionate burden.

Beneficence, non-maleficence and autonomy. Given that prospective parents are already explicitly assuming a parental role identity, they have special duties of procreative beneficence towards their future child and should first optimize their folic acid levels. Potential parents of category 4 – sexually active but leaving possible conception up to chance – also have an elevated moral duty. They should either start using contraceptives or else optimize their folic acid levels. Concerning potential parents who use contraception, some PCC advocates argue that the packages of birth control pills should advise that upon stopping with birth control pills in order to try to conceive, one should immediately switch to folic acid supplements [1]. On our analysis, such initiatives are warranted. Moreover, this advice could be broadened to include the information that, given the high incidence rates of unplanned pregnancy, any (presumably) fertile and sexually active woman (i.e. not only those in category 5 but also those in categories 4 and 3) should consider optimizing her folic acid level to decrease the risk of neural tube defects. Persons in the other categories are so far removed from a potential conception that they have no duty of preconception beneficence to take folic acid.

### Obesity

The potential adverse pregnancy outcomes brought on by conception and gestation in an overweight body can be severe (increasingly so as one moves towards actual (morbid) obesity). Paraphrasing the synopsis of several systematic reviews provided by the Health Council of The Netherlands [1], compared to women of normal weight (BMI between 20 and 25), for obese women (BMI 30<) the risk of diabetes is increased by a factor of 1.4 to 20, the risk of hypertension by 2.2 to 21.4, and the risk of pre-eclampsia by 1.2 to 9.7. These factors increase the risk of harming the foetus, making the incidence of neural tube defects rise by a factor of 1.5 to 3.0 times in children of obese mothers and the risk of stillbirth by a factor of 2.5 to 3.4. These risks are also elevated, albeit to a lesser degree, for overweight persons (BMI 25–30). A clear solution to reduce these risks would be the timely optimization of one’s body weight.

Given the framework of preconception responsibility outlined above, should any normal, reasonably prudent potential parent normalize her body weight before attempting pregnancy or if there is a risk of an unplanned pregnancy?

Cognizance. In contrast to public knowledge on folic acid, it is widely known that abnormally high body weight is related to a host of health problems. However, the link between body weight and health problems of potential future offspring is likely to be substantially less well-known. For instance, without scientific knowledge on the issue, some might even speculate that being overweight may provide a better, more nurturing conceptive and gestational environment.

Control. Reducing and/or substantially changing the nature of one’s food intake can be very demanding to many people, for reasons of individual psychology, group psychology, (financial) access to healthy food, etc. It will often require a trying expenditure of time, effort and possibly money. In some cases, problematic body weight is not (or not primarily) the result of one’s behaviour, but a largely inescapable outcome of a genetic constitution, a medical condition, or a medication regime. Case by case, and risk group by risk group, these factors should be taken into account in the calculus of personal responsibility. That said, many overweight persons are in a position to optimize their body weight.

Causation. Being overweight prior to conception can causally contribute to several forms of adverse pregnancy outcomes [1]. To the extent that it is the overweight persons’ acts and/or omissions that causally brought about their risk-increasing body weight, they open themselves up to being held morally accountable for exposing their potential child to the attendant risks. However, considerations of proportionality might substantially relax, if not absolve them of, such moral accountability.

Proportionality. For several reasons, it would be problematic to make the moral demand on overweight potential parents to suspend all attempts at conception until they have successfully optimized their weight. For instance, the weight-optimizing enterprise might take so much time for certain persons that, by the time they reach an optimal weight, other obstacles have come into play (e.g. maternal age over 35, loss of a willing reproductive partner, etc.). Moreover, persons burdened by a relative lack of financial resources or by certain genetic or medical conditions may find it virtually impossible to optimize their body weight, or doing so may be disproportionally difficult for them. Therefore, it would be problematic to demand compliance. Rather, only a proportionate, sustained effort to optimize one’s weight can reasonably be demanded [23]. Although fertile persons with weight problems could disregard all directive messages and simply go ahead and conceive, that would constitute a (legally permissible yet) morally tainted exercise of their reproductive liberty.

Beneficence, non-maleficence and autonomy. Prospective parents, already having a future child in view, would also need to invest such effort out of their duty of procreative beneficence. For potential parents of category 4, who are leaving it up to chance if they get pregnant/impregnate, a heightened moral imperative to keep their body weight under control also holds. Considering the fact that tackling overweight will often be a much more demanding task than taking folic acid, other types of potential parents – who have only a lesser or no duty of beneficence – should only be non-directively informed about the risks to future children of preconception overweight, for, in view of the demandingness, the proportionality calculus would allow more leeway to the potential parents’ lifestyle choices or habits over their duty of non-maleficence.

### Genetic screening

A great number of diseases and handicaps are rooted in one’s genetic make-up. Increasingly, potential parents can find out whether they are carriers of genetic factors that significantly increase the probability of adverse pregnancy outcomes, most commonly for autosomal dominant or autosomal recessive disorders, for which there is, respectively, a ½ or ¼ chance of producing the disorder in one’s offspring.

Given the framework of preconception responsibility outlined above, should any normal, reasonably prudent potential parent undergo genetic screening before attempting to conceive?

Cognizance. Basic knowledge about genetic risks clearly remains an issue about which more public health education is needed [24]. The same holds a fortiori for the additional awareness that genetic screening prior to conception is available and might be helpful. However, public knowledge levels on these issues seem likely to increase given the emergence of public campaigns on PCC and on genetic literacy, as well as the publicity campaigns by commercial (quasi-)direct-to-consumer genetic testing companies.

Control. The Health Council of The Netherlands argues that “the scenario must be avoided in which a decision not to make use of a service such as preconceptual carrier screening is regarded as irresponsible”, based in part on the consideration that one’s genetic constitution is not a ‘controllable’ factor in the sense that for instance one’s overweight or one’s folic acid level are ‘controllable’ [1]. However, even though one cannot exercise any meaningful control over one’s genetic constitution, in many cases one can exercise meaningful control over how one will expose one’s future offspring to risks stemming from it.

Causation. Though one is not oneself the cause of one’s genetic constitution and thus must surely not be blamed or in any way judged for it, one can become the cause of an adverse condition in one’s offspring due to one’s unwillingness to undertake genetic screening.

Proportionality. How should we map the benefit/burden calculus for genetic screening? On the benefit side, the amount of suffering one can avoid is significant, as shown for instance by the Cypriot campaign against beta-thalassemia [25]. Equally, the degree of certainty that one will effectively avoid significant suffering can often be high, for instance when one has been diagnosed with a dominant or recessive autosomal disorder, or when one is a member of a population with an elevated risk, e.g. 1 in 30 Dutch persons is a carrier of cystic fibrosis [1]. On the burden side, undergoing genetic carrier screening demands very little of a potential parent: providing a blood or sputum sample or even only a buccal swab. The burdens rather lie in handling knowledge regarding one’s genetic status (which may reveal much more than just the risks for one’s future offspring, namely risks to oneself and to one’s genetic relatives). To avoid such burdens, one may want to invoke a ‘right not to know’. Another set of substantial burdens pertains to the affliction-avoiding interventions one may have to engage in when a substantial genetic risk has been found (e.g. the strains of undergoing IVF/PGD cycles). Moreover, in regions without publicly subsidized health care for these purposes, both the testing itself and the ensuing interventions can be extremely costly for potential parents. Then again, when one takes into account the potentially astronomical costs to a person of living with a severe affliction, plus the costs of (lifelong) care for severely afflicted persons, even high costs of tests and interventions may nonetheless be relatively proportionate. A normal and reasonably prudent prospective parent (i.e. category 5), who has good reason to assume that he/she belongs to a group with an elevated genetic risk of severely afflicting future offspring, would be acting morally irresponsibly if he/she knowingly foregoes genetic carrier screening.

Beneficence, non-maleficence and autonomy. In Cyprus, persons who want to marry before the Cypriot Orthodox Church (and who can be reasonably expected to try to bear children) are obliged to first have their carrier status for beta-thalassemia checked [25]. On our analysis, such a scheme seems to be based on a proper conception of preconception beneficence. All prospective parents (i.e. those in category 5) whose genetic predicament is known to be analogous to that of the Cypriots can reasonably be expected to engage in genetic screening for their respective risk factors. In another scheme proposed by the UK Human Genetics Commission, population-wide genetic screening for a variety of genetic risks would be organized during the final years of the secondary education system [4]. According to this proposal, adolescents should be merely informed in an entirely non-directive way of the possibility of being screened and about what screening can achieve. One might argue that a large-scale implementation of genetic screening would inadvertently give rise to some implicit directivity. Yet on our analysis, within proper bounds, such awareness-raising concerning the preconception responsibilities of potential parents in categories 1, 2, 3, and certainly 4, may be justified. For instance, to those in categories 1 and 2, one could already mention the moral importance of avoiding severe afflictions in one’s future children, and leave it to their own discretion to think about the (dis)proportionality of preemptively investigating their genetic risk factors. For potential parents in category 3 and certainly to those in category 4, one could both heighten awareness of the likelihood of unplanned pregnancy and signal the importance (made more acute in view of their coital activity) of getting to know their genetic risk profiles. A similar conclusion can be reached starting from a discussion of reproductive autonomy [26]. It must be noted, however, that none of this would compromise the right of a potential parent to conscientiously object, their right to exercise the right not to know, or their right to reproduce.

## Summary

We began this paper by briefly sketching the state of the art and the state of the debate regarding PCC. We explained how the PCC paradigm can enlist all sorts of ‘potential parents’ in its preventative project by imposing some form of preconception responsibility upon all of them. This identification of large swathes of society as some kind of potential parent seems to entail a real risk of a ‘responsibility explosion’. If one maps these categories out on the lifespan of a single person, most people would have to assume at least some minimal form of PCC responsibility during their entire period of fertility. This situation seems to be further aggravated by the increasing number of PCC measures that are becoming available and by the ‘capability imperative’ they inevitably bring about. Given these substantial burdens, we have attempted to develop a preliminary framework of preconception responsibility that identifies preconception responsibilities in a sufficiently specific way. To that end, we have applied a theory of moral responsibility, involving principles of (preconception) beneficence, (preconception) non-maleficence and individual autonomy, to the cases of folic acid, obesity and genetic screening.

Our discussion of PCC has been primarily restricted to potential parents. Further work, seeking to develop a comprehensive rather than a preliminary ethical framing of PCC such as the one offered here, needs to take into account much broader socio-political realities and normative frameworks. Indeed, an in-depth analysis would also need to investigate the PCC responsibilities of medical professionals, health care institutions, the potential parent’s government, employer, and cultural, social and family communities. Our focus on potential parents is by no means intended to detract from the responsibilities of the other actors and institutions in the field of PCC.

We have argued that prospective parents as well as several other categories of potential parents have at least a minimal moral duty to *sufficiently try to optimize* the circumstances of conception. Although we have sought to apply only a ‘minimal’ standard (i.e. one that prescribes a ‘moral minimum’), circumstances may conspire to make the commitment to only ‘minimal’ duties of PCC overly burdensome nonetheless. That would be a sufficient reason to reject even some of such ‘minimal’ duties. It would certainly be absurd to argue that an agent X has a duty Y, if X is irremediably incapable of meeting duty Y. Similarly, it would be unreasonable to expect from potential parents that they perform supererogatory acts of PCC.

There are many cases in which realizing one’s basic moral duties in no more than a minimally sufficient way may in practice require sustained attentiveness over a long period of time, as well as intensive effort and substantial sacrifice of self-centred activity. The current armoury of PCC has not yet amassed to such a dramatic extent that the default, responsible way to procreate would require the use of artificial reproductive technologies as Bill Handel would have it – indeed it seems highly doubtful that such a scenario would ever come about. Nevertheless, it will probably already be hard for many people today to adequately discharge themselves of the minimal PCC duties advocated here. The strains involved will only increase as new effective means of PCC interventions are made available. The strains themselves, however, should not be invoked as an argument against PCC, as long as a normal potential parent of reasonable prudence can be expected to bear such strains in order to reduce the likelihood of serious adverse pregnancy outcomes.

## Abbreviations

PCC: Preconception care; PB: Procreative beneficence.

## Competing interests

The authors declare to have no competing interests. This study was funded by research grant G.0214.10 provided by the Research Foundation Flanders (FWO).

## Authors’ contributions

PB prepared and edited the article drafts. GP and SS commented on and extended the drafts. SS edited the final version. All authors read and approved the final manuscript.

## Pre-publication history

The pre-publication history for this paper can be accessed here:

http://www.biomedcentral.com/1472-6939/15/5/prepub
